# Inorganic polyphosphate as an energy source in tumorigenesis

**DOI:** 10.18632/oncotarget.27838

**Published:** 2020-12-15

**Authors:** Jerusha Boyineni, Simone T. Sredni, Naira V. Margaryan, Lusine Demirkhanyan, Michael Tye, Robert Johnson, Fernando Gonzalez-Nilo, Mary J.C. Hendrix, Evgeny Pavlov, Marcelo B. Soares, Eleonora Zakharian, Sergey Malchenko

**Affiliations:** ^1^Department of Cancer Biology & Pharmacology, University of Illinois College of Medicine, Peoria, Illinois, USA; ^2^Department of Surgery, Feinberg School of Medicine, Northwestern University, Chicago, Illinois, USA; ^3^Division of Pediatric Neurosurgery, Ann & Robert H. Lurie Children’s Hospital of Chicago, Chicago, Illinois, USA; ^4^Department of Biochemistry, Robert C. Byrd Health Sciences Center and Cancer Institute, West Virginia University, Morgantown, West Virginia, USA; ^5^Center for Bioinformatics and Integrative Biology, Universidad Andres Bello, Santiago, Chile; ^6^Centro Interdisciplinario de Neurociencia de Valparaíso, Facultad de Ciencias, Universidad de Valparaíso, Valparaíso, Chile; ^7^Department of Biology, Shepherd University, Shepherdstown, West Virginia, USA; ^8^Department of Molecular Pathobiology, New York University, College of Dentistry, New York, New York, USA; ^*^These authors contributed equally to this work

**Keywords:** polyphosphate, energy source, OXPHOS, glycolysis, metabolism

## Abstract

Cancer cells have high demands for energy to maintain their exceedingly proliferative growth. However, the mechanism of energy expenditure in cancer is not well understood. We hypothesize that cancer cells might utilize energy-rich inorganic polyphosphate (polyP), as energetic reserve. PolyP is comprised of orthophosphates linked by phosphoanhydride bonds, as in ATP. Here, we show that polyP is highly abundant in several types of cancer cells, including brain tumor-initiating cells (BTICs), i.e., stem-like cells derived from a mouse brain tumor model that we have previously described. The polymer is avidly consumed during starvation of the BTICs. Depletion of ATP by inhibiting glycolysis and mitochondrial ATP-synthase (OXPHOS) further decreases the levels of polyP and alters morphology of the cells. Moreover, enzymatic hydrolysis of the polymer impairs the viability of cancer cells and significantly deprives ATP stores. These results suggest that polyP might be utilized as a source of phosphate energy in cancer. While the role of polyP as an energy source is established for bacteria, this finding is the first demonstration that polyP may play a similar role in the metabolism of cancer cells.

## INTRODUCTION

Evolutionarily, the eukaryotic genome resulted from endosymbiosis between the progenitor of the eukaryotic lineage and an aerobic proteobacterium–the origin of mitochondria [1–3]. During nutritional stress or hypoxia, which also are common factors in tumorigenesis, aerobic bacteria accumulate a ubiquitous polymer-inorganic polyphosphate (polyP), which contains high-energy phosphoanhydride bonds - and uses it as a steady source of energy [4]. The unique advantage of polyP as an energy source is that it can be directly converted into ATP without any intermediate steps and it does not require oxygen to generate energy [5]. Earlier seminal works by Arthur Kornberg demonstrated that inorganic polyphosphate plays critical roles in several organisms, particularly in bacteria and yeast [6–10]. In the recent years, a plethora of important functions of this homopolymer have been highlighted in the context of various mammalian systems [11–21]. Among numerous functions of polyP, one of the most evolutionary fascinating is its contribution to energy metabolism [15, 22–24]. In higher eukaryotes, polyP is involved in regulation of different mitochondrial functions [25], including regulation of intracellular ATP [22]. It was shown recently that incubation of synthetic polyP with human osteogenic sarcoma cells led to accumulation of ADP and ATP in the extracellular space of the cells [26]. However, many aspects of polyP function in mammalian organisms are not completely understood.

Our present findings provide a novel insight into the abundance and accumulation of polyP in different types of cancer, suggesting a possible common role of this polymer in tumorigenesis. In particular, our study shows that polyP could be utilized in these cells as a source of phosphate energy.

## RESULTS

### Accumulation of polyP in cancer cells of different origin

Recently we developed a derivation method of human Radial Glial (RG) cells (see below: LC26-10R and LCAS-7R cell lines)-the progenitor cells for adult neural stem cells-that is based on spontaneous neural differentiation of human induced pluripotent stem cells [27]. We also described an animal model of malignant childhood brain tumors that can be generated by orthotopic transplantation of RG cells into the sub-ventricular zone of the mouse brain [28]. Consequently, we derived and characterized brain cancer stem cells (CSCs), also known as BTICs, from these model tumors (see below: LC26-RTL(4) and LCAS-7RTL(138) cell lines) [29].

It is well established that uncontrolled proliferation of CSCs, the cells responsible for tumor initiation and maintenance, requires an abundant supply of energy [30, 31]. However, the mechanism by which cancer cells maintain their high demands of energy expenditure is not well understood. We hypothesized that cancer cells utilize some unconventional molecules as their energetic reserve. This hypothesis pointed our attention to a highly conserved homopolymer that possesses numerous high-energy phosphoanhydtide bonds, i.e., inorganic polyphosphate. Considering the ubiquitous nature of polyP, along with the cancer cell high-energy demand and metabolic plasticity [31], it is conceivable that polyP might be a potential component of the cancer cell energy supply [32].

**Figure 1:**
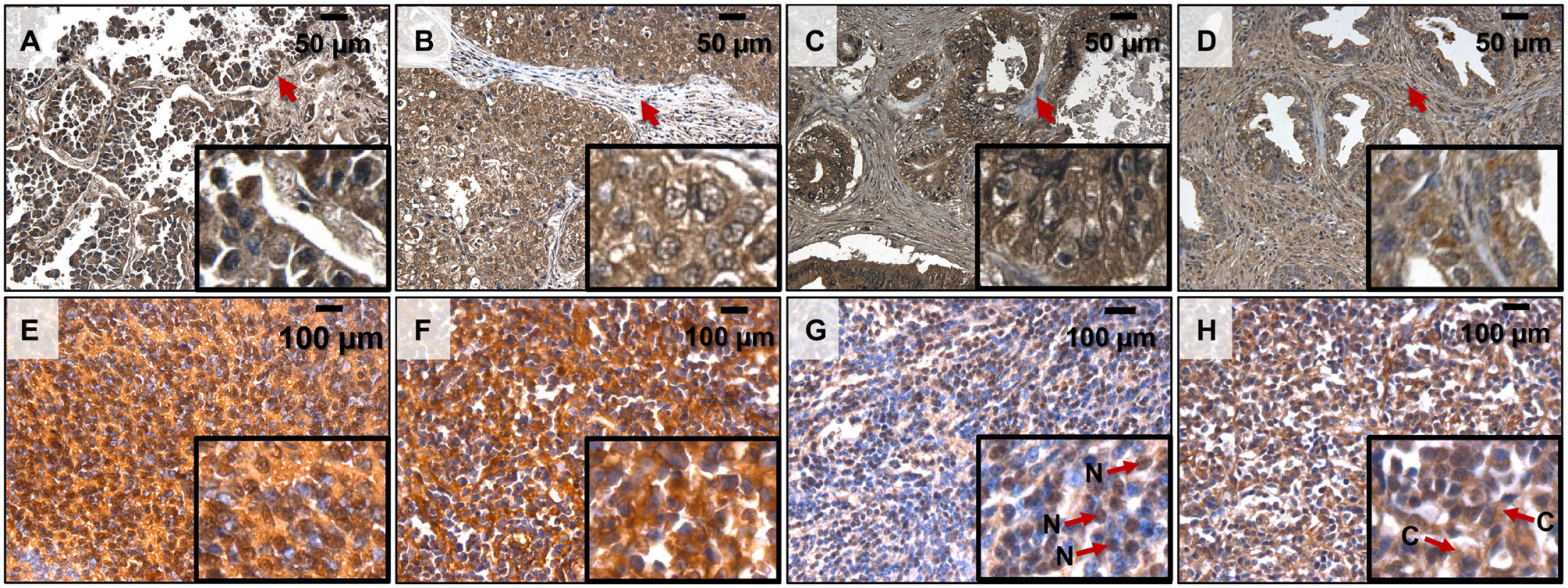
PolyP detection by immunohistochemistry in human cancer samples. (**A**) Lung Bronchoalveolar Adenocarcinoma 20× (16× digital magnification) (TMA MC484 A2); (**B**) Breast Invasive Ductal Adenocarcinoma 20× (16× digital magnification) (TMA MC484 A4); (**C**) Small Intestinal Adenocarcinoma 20× (16× digital magnification) (TMA MC484 A6); (**D**) Prostate Adenocarcinoma 20× (16× digital magnification) (TMA MC484 C5), (**E**) Medulloblastoma 40× (16× digital magnification) (TMA GL 1001 I6); (**F**) Medulloblastoma 40× (16× digital magnification) (TMA GL 1001 I7); (**G**), Medulloblastoma 40× (16× digital magnification) (TMA GL 1001 I3); (**H**) Medulloblastoma 40× (16× digital magnification) (TMA GL 1001 I2). PolyP identified as dark brown precipitates. Arrows in panels a-d indicate polyP negative fibroblasts within the tumors. Arrows in panels g, h indicate polyP signal localization in Nuclei (N) or Cytoplasm (C).

Remarkably, in line with our hypothesis, we detected a high signal of polyP in a broad array of primary tumor types, including human bronchioloalveolar adenocarcinoma, invasive ductal adenocarcinoma, small intestine adenocarcinoma, prostate adenocarcinoma, and medulloblastoma (Figure 1). As the polyP staining requires multiple steps, which potentially could lead to removal of the polymer and decrease of the signal, instead of applying the staining to a matching tissue as a negative control, we used non-transformed cells in a tumor bulk as a reference to point out cancer-related polyP accumulation. Interestingly, in the adenocarcinomas the polyP staining was observed primarily in the cytoplasm of malignant epithelial cells, while in the medulloblastomas it was observed either in both nuclei and cytoplasm (Figure 1E), in the nuclei (Figure 1G), or in the cytoplasm (Figure 1F and 1H) of the neoplastic cells.

**Figure 2:**
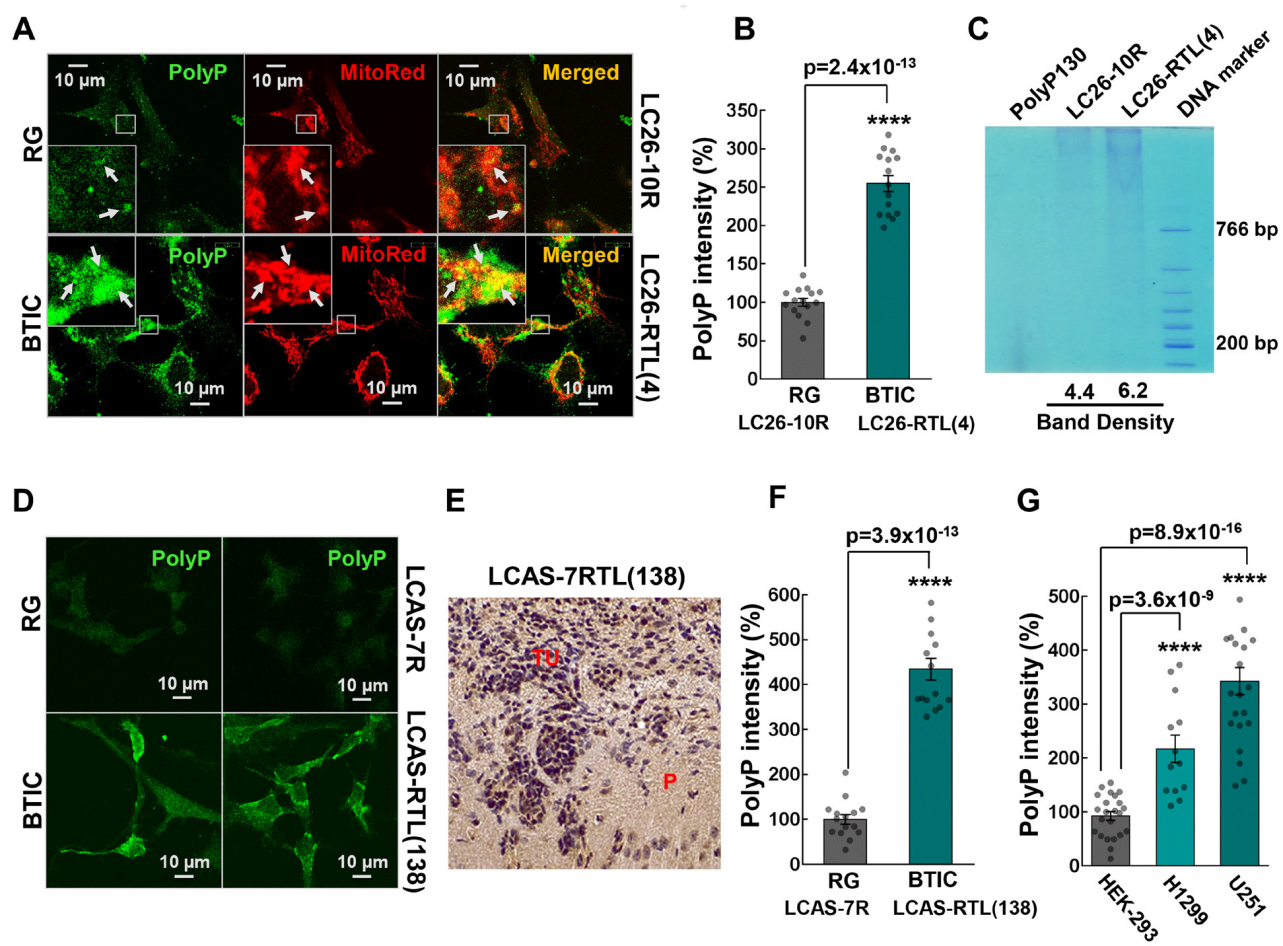
(**A**) Colocalization of polyP with mitochondria of LC26-10R (RG) and LC26-RTL(4) (BTIC) cells. PolyP was detected by immunocytochemistry using polyP-binding domain (PPBD) affinity labeling (green). Mitochondria were stained with MitoTracker Red CMXRos. (**B**) Intensities of polyP signal in LC26-10R vs. LC26-RTL(4). (**C**) polyP extraction from LC26-10R (RG) and LC26- RTL(4) (BTIC) cells along with quantification of band density signal. (**D**) Representative fluorescent images of polyP labeling of LCAS-7R (RG) and LCAS-7RTL(138) (BTIC) cells. (**E**) DAB staining of polyP in LCAS-7RTL(138) generated tumor (2 month post-inoculation into NOD-SCID mice brain). P parenchyma; TU tumor mass. (**F**) Intensities of polyP signal in LCAS-7R (RG) vs. LCAS-7RTL(138) (BTIC). (**G**) Intensities of polyP signal in HEK-293 *vs.* H1299 lung cancer and U251 glioma cells.

The analysis of polyP content in the RG and BTICs revealed significantly elevated levels of the polymer in BTICs in comparison to the corresponding control RG lines (Figure 2A, 2B, 2D and 2F). Deciphering the intracellular localization of the polymer showed that polyP intensely, but not exclusively, resides in mitochondria and its proximity. A direct extraction of polyP [33] further demonstrated increased polyP content in the cancer cell line (Figure 2C). Moreover, we found that polyP is increased in the derived BTIC tumors (Figure 2D–2F), and cancer cell lines H1299 and U251 (Figure 2G).

### Utilization of polyP in cancer cells during starvation and ATP depletion

**Figure 3:**
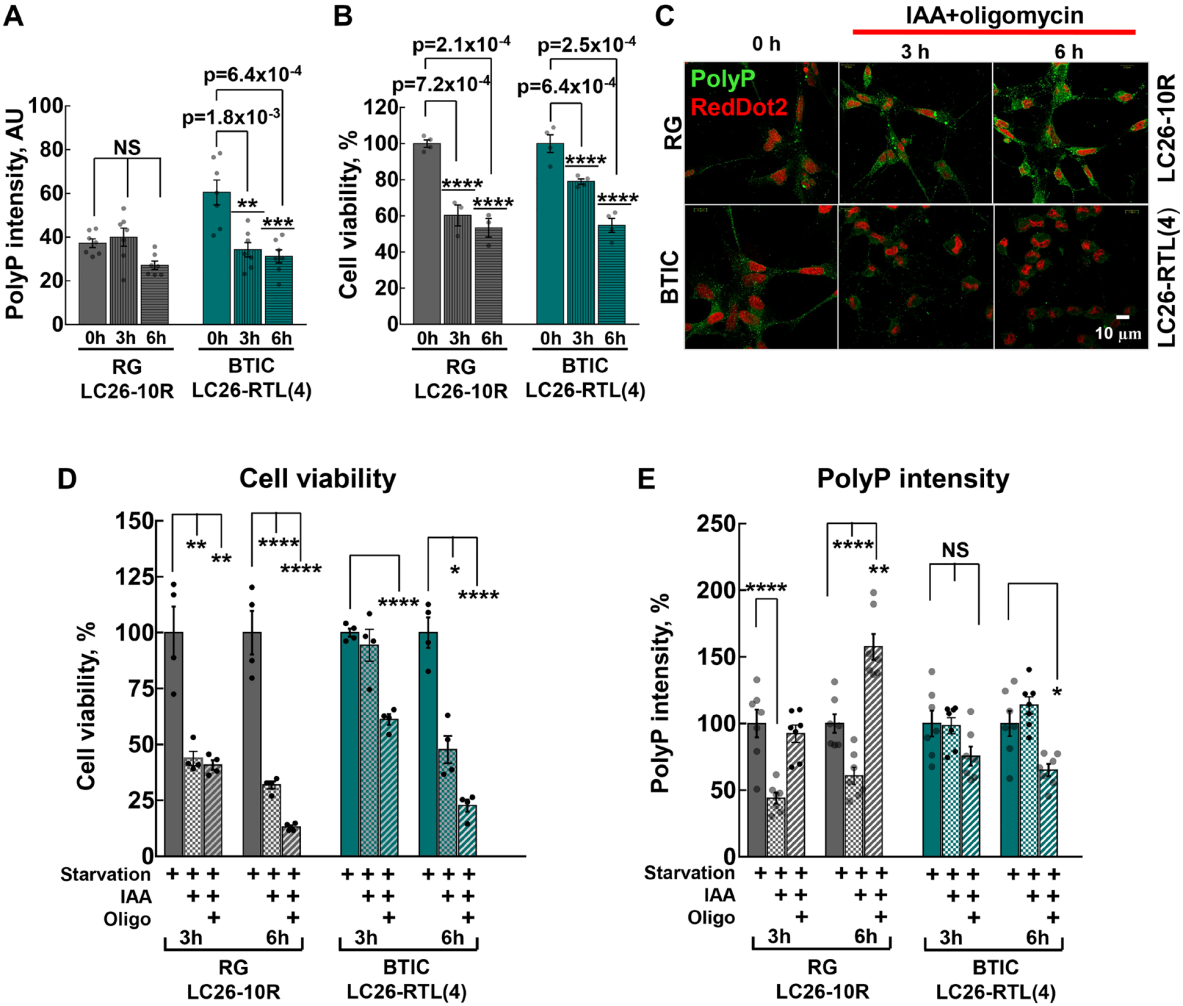
(**A**) Intensity of cellular polyP fluorescence signal before and after 3 hour and 6 hour starvation of LC26-10R (RG) (*p* = 1.0 for 0 h vs. 3 h starvation, and *p* = 0.06 for 0 h vs. 6 h starvation) and LC26- RTL(4) (BTICs) (*p* = 1.8 × 10^-3^ for 0 h vs. 3 h starvation, and *p* = 6.4 × 10^-4^ for 0 h vs. 6 h starvation). Glucose deprivation is indicated as starvation. (**B**) Cell viability before and after 3 hour and 6 hour starvation of LC26-10R (RG) (*p* = 7.2 × 10^-4^ for 0 h vs. 3 h, and *p* = 2.1 × 10^-4^ for 0 h vs. 6 h) and LC26- RTL(4) (BTICs) (*p* = 6.4 × 10^-4^ for 0 h vs. 3 h, and *p* = 2.5 × 10^-4^ for 0 h vs. 6 h). (**C**) PolyP staining (green) before and after 3 hour and 6 h starvation along with glycolysis inhibitor IAA and mitochondrial ATP-synthase (OXPHOS) inhibitor olygomycin treatment of RG and BTICs. RedDot2 (red) nuclear staining. (**D** and **E**) Cell viability and intensity of polyP fluorescence signal in RG and BTICs after 3 h and 6 h starvation, IAA or IAA/Oligomycin treatment. d, RG (*p* = 1.04 × 10^-2^ for 3 h starvation vs. 3 h starvation+IAA, *p* = 1.09 × 10^-2^ for 3 h starvation vs. 3 h starvation+IAA+Oligo, *p* = 3 × 10^-3^ for 6 h starvation vs. 6 h starvation+IAA and *p* = 3.7 × 10^-3^ for 6 h starvation vs. 6 h starvation+IAA+Oligo); and BTICs (*p* = 0.57 for 3 h starvation vs. 3 h starvation+IAA, *p* = 3.9 × 10^-3^ for 3 h starvation vs. 3 h starvation+IAA+Oligo, *p* = 6 × 10^-4^ for 6 h starvation vs. 6 h starvation+IAA and *p* = 2.3 × 10^-3^ for 6 h starvation vs. 6 h starvation+IAA+Oligo). (E) RG (*p* = 5.5 × 10^-5^ for 3 h starvation vs. 3 h starvation+IAA, *p* = 0.8 for 3 h starvation vs. 3 h starvation+IAA+Oligo, *p* = 2.7 × 10^-5^ for 6 h starvation vs. 6 h starvation IAA and *p* = 1.1 × 10^-3^ for 6 h starvation vs. 6 h starvation IAA+Oligo) and BTICs (*p* = 0.8 for 3 h starvation vs. 3 h starvation+IAA, *p* = 0.06 for 3 h starvation vs. 3 h starvation+IAA+Oligo, *p* = 0.31 for 6 h starvation vs. 6 h starvation+IAA and *p* = 5.8 × 10^-3^ for 6 h starvation vs. 6 h starvation+IAA+Oligo).

It has long been established that during nutritional deprivation, aerobic bacteria accumulate polyP and use it as a steady source of energy [34, 35], utilizing its high-energy phosphoanhydride bonds as a substrate for generation of ATP from ADP [36]. To elucidate the role of polyP in energy metabolism we used extensively studied BTICs and RG cell lines. As cancer stem cells, including BTICs, predominantly derive their energy from glycolysis [29–31], our next strategy was to determine whether cancer or their control cells utilize polyP under glucose deprivation (starvation) conditions. The decreased glucose intake ensured down-regulation of any glucose-dependent energy pathways, particularly glycolysis. We found that, in striking contrast to RG cells, where the brief starvation (up to 6 hours) did not triggered any significant impact on polyP levels, the levels of the polymer markedly (> 50%) declined in cancer cells (Figure 3A). In addition to starvation, the suppression of ATP synthesis by inhibiting both glycolysis and mitochondrial ATP-synthase (OXPHOS) decreased levels of polyP in BTICs even further, while in RG the levels of the polymer became highly accumulated after the same treatment (6 hours) (Figure 3C and 3E). Noteworthy, the utilization of polyP in BTICs correlated with their higher survival rates, compared to RG (Figure 3A, 3B, 3D and 3E). Interestingly, suppression of glycolysis without inhibition of mitochondrial ATP-synthase significantly decreased levels of polyP in RG (Figure 3E). These results suggest that mitochondrial ATP-synthase is involved in polyP metabolism in RG cells, further highlighting the difference in polyP turnover between normal and cancer stem cells.

### Enzymatic hydrolysis of polyP impairs cell viability and depletes the ATP pool in cancer cells

**Figure 4:**
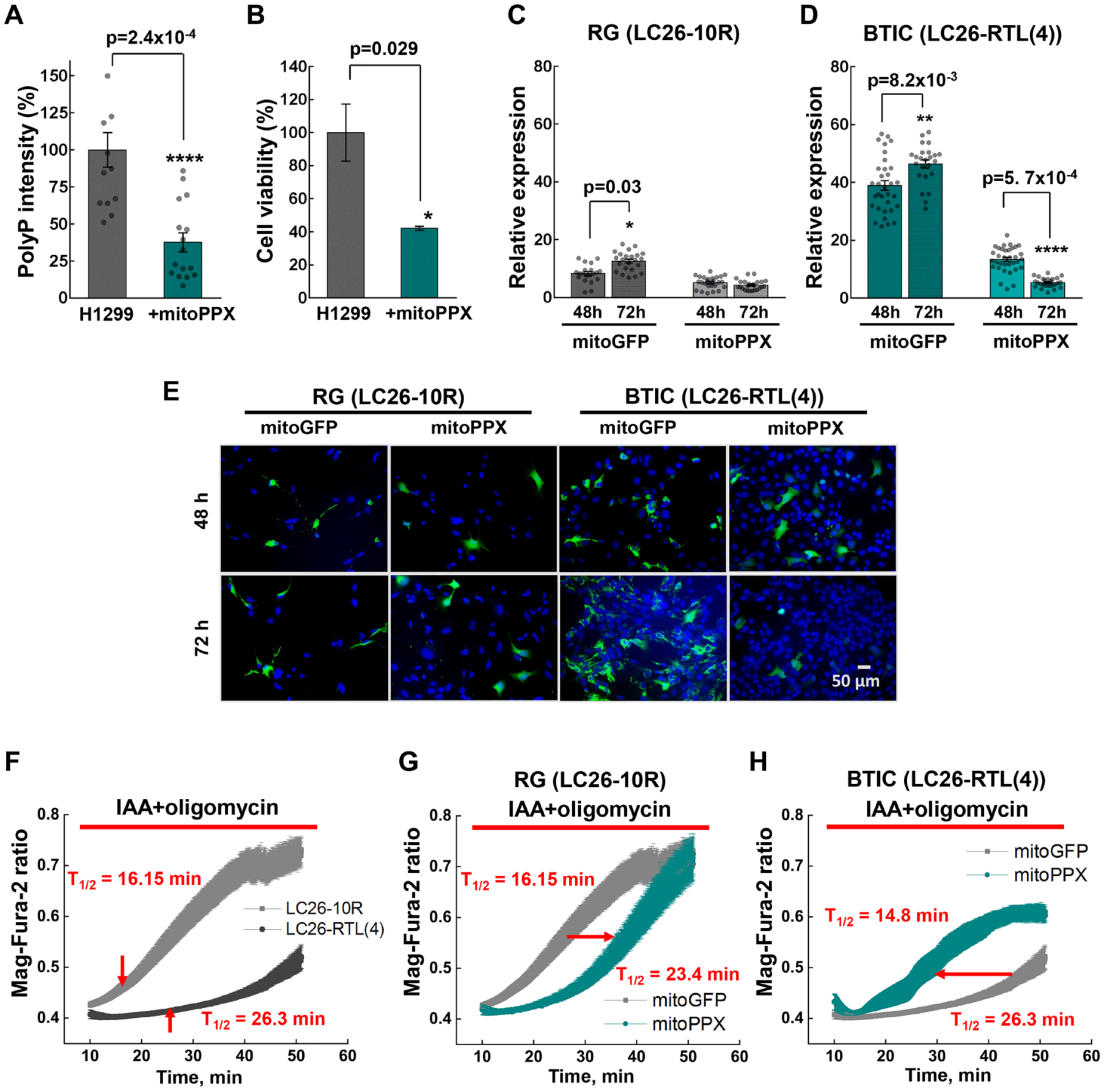
(**A**) PolyP intensities of H1299 cells transiently expressing mitoPPX. (**B**) Cell viability of H1299 lung cancer cells upon transient expression of mitoPPX, obtained by MTS assay. (**C** and **D**) Relative expression of mitoGFP or mitoPPX-GFP in LC26-10R and LC26- RTL(4) cells 48 hour and 72 hour post-transfection. (**E**) Representative images of LC26-10R and LC26- RTL(4) cells transiently expressing mitoGFP or mitoPPX-GFP after 48 hour and 72 hour post-transfection : green signal – GFP alone or GFP-PPX; blue – DAPI nuclear staining. (**F**) Comparative fluorescence measurements of the intracellular Mg^2+^ concentration performed on LC26-10R and LC26- RTL(4). (**G** and **H**) Fluorescence measurements of the intracellular Mg^2+^ concentration performed on LC26-10R (G) and LC26- RTL(4) (H) transiently expressing mitoPPX (cyan) vs. control cells. Intracellular Mg^2+^ increases as ATP is hydrolyzed in control and mitoPPX cells in response to inhibitors of ATP production, IAA (20 μM) and oligomycin (20 μM).

Next, we assessed whether depletion of polyP exerts an effect on cancer cells energy metabolism. Using an enzyme that specifically and sequentially cleaves polyP from its terminal phosphate residue, yeast exopolyphosphatase (PPX), we first evaluated the degree of polyP hydrolysis, and then its effect on cell viability. Since we detected high levels of polyP in mitochondria (Figure 2A), we used a construct that specifically targets PPX expression in this organelle (mitoPPX) [22, 36]. Transiently expressing mitoPPX in H1299 lung cancer cells markedly reduced levels of polyP and cell viability (Figure 4A and 4B). In line with these results, transient expression of mitoPPX in BTICs significantly compromised the viability of BTICs in a time-dependent manner, compared to the RG (Figure 4C–4E).

Next, we questioned whether high amounts of polyP detected in tumor cells contribute directly to their energy supply. To assess the role of polyP in this paradigm, we estimated the speed of ATP utilization in the presence or absence of mitoPPX upon inhibition of ATP production by inhibiting glycolysis with iodoacetic acid (IAA) and mitochondrial ATP-synthase with oligomycin.

We found that unlike RG, the time of ATP consumption in cancer cells was dramatically longer (T_1/2_ = 16.1 ± 1.09 min in RG vs. T_1/2_ = 26.3 ± 0.54 min. in TL) (Figure 4F). Deprivation of mitochondrial polyP storage using mitoPPX resulted in a prolonged ATP consumption in RG, which might be due to an unknown compensatory mechanism (Figure 4G). In contrast, depletion of polyP in cancer cells essentially shortened their rates of ATP utilization, decreasing the T_1/2_ time from 26.3 ± 0.54 to 14.8 ± 0.44 min (Figure 4H). These results suggest that polyP is utilized as a source of phosphate energy in BTICs, prolonging the time required for a complete energetic collapse when known sources of ATP are blocked.

**Figure 5:**
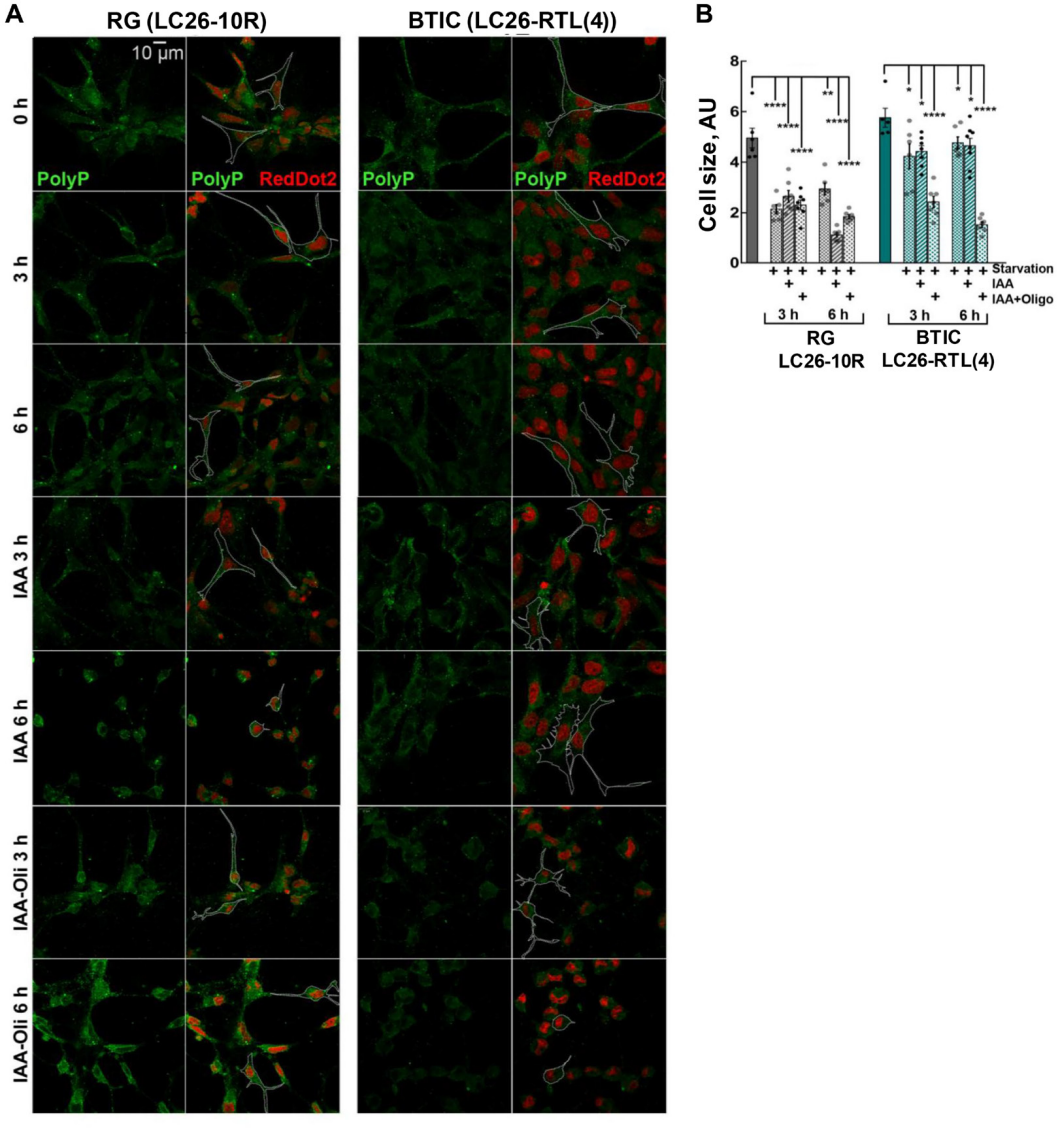
(**A** and **B**) Morphology and cellular size of LC26-10R (RG) and LC26- RTL(4) (BTICs) after 3 h and 6 h starvation, IAA, or IAA/Oligomycin treatment. PolyP staining (green). RedDot2 (red) nuclear staining.

Cell survival rates under starvation conditions (lack of growth factors and nutrients) are contingent upon utilization of all intracellular energy reservoir molecules. Excessive deprivation of exogenous and endogenous energy sources eventually impacts cell size and morphology [37]. Therefore, apart from ATP consumption rates, we also estimated morphological changes to the cells upon their nutritional and energetic deprivation. Indeed, we noticed that different treatment conditions distinctly impacted the shape and size of the cancer and the control cells. Systematically assessing the alterations in shape and size of the treated cells, we detected essential differences in cellular morphology between RG and BTICs (Figure 5A and 5B). Notably, a significant decline in polyP intensity in cancer cells upon the inhibition of ATP production with IAA and oligomycin treatment (Figure 3E) was also accompanied by a progressive cellular atrophy and, importantly, by a dramatic alteration in cell morphology (Figure 5A and 5B). Despite the fact that deprivation of ATP sources impacted the control RG cells in general, they did not show significant difference between the different treatments and time kinetics. On contrary, the cancer BTICs cells showed a strong time-dependent decline in their size and morphology upon the inhibition of both ATPase and glycolysis (Figure 5B). These results suggest that in cancer cells polyP might, in addition to its role of an energy fuel, also be directly or indirectly a contributing factor in supporting cellular infrastructure.

## DISCUSSION

Despite enormous efforts searching for new therapeutic targets and great progress in patient survival, cancer remains one of the most deadly human diseases [38], primarily due to the limited number of effective cancer-specific therapies. One of the main challenges in the field is to identify cancer-specific targets that play indispensable roles in different forms of cancer. The switch from mitochondria oxidative metabolism to alternative energy sources is key in the cancer energy supply. Recognizing that evolutionarily mitochondria originated from aerobic proteobacterium [1–3], and that polyP is a ubiquitous polymer, we hypothesized that polyP may play a role in cancer cells as an energy source [22, 32], similar to its role in bacteria [34, 35] during starvation or hypoxia.

Our findings provide a novel insight into the presence of polyP in different types of cancer, suggesting a possible common role of this polymer in tumorigenesis (Figure 1). Since we used qualitative immunohistochemical assessment of the polyP presence, highlighting the expression (yes or no) and location (nuclei or cytoplasm) of the polymer in the studied tissue sections, it will be interesting to conduct a comparative analyses of polyP accumulation in different types of cancer. The distinctive benefit of polyP as an energy source is that it could be directly converted into ATP without any intermediate steps and it does not require oxygen [5]. This function would be particularly important during periods of nutritional or hypoxic stress in microenvironments of aggressively growing tumors. Significant reduction of polyP levels during brief starvation in BTICs is in line with this premise (Figure 3A), as well as our finding that increased consumption of polyphosphate during the starvation correlates with increased survival of BTICs (Figure 3B). Similar effect was observed at the early hours (3 h) of starvation and suppression of ATP production (Figure 3D and 3E). However, the correlation become insignificant after 6 hours of the treatment and cell viability drastically diminished in both RG and BTICs regardless of the polyP levels. It indicates that if polyP is indeed utilized by cancer cells as an additional source of energy it may only briefly delay an energetic collapse once the cells lost glycolysis and OPHXOS as their main sources of ATP.

Enzymatic hydrolysis of polyP significantly compromised the viability of BTICs in a time-dependent manner (Figure 4), indicating an important role of polyP in cancer cell homeostasis and highlighting the polymer as potentially effective drug target in a wide range of different tumor types. The hydrolysis also drastically impacted ATP consumption pattern in BTICs (Figure 4F–4H), thus, supporting our hypothesis of polyP utilization as a source of phosphate energy in cancer cells. Interestingly, we found that starvation did not significantly impact polyP levels in RG. Remarkably, the combination of starvation and suppression of glycolysis did force the cells to utilize polyP, while additional suppression of mitochondrial ATP-synthase significantly increased the levels of the polymer, suggesting that the protein complex is involved in polyP metabolism in RG cells. Most recent study revealed that mitochondrial ATP-synthase might be a key enzyme implicated in polyP biogenesis [15].

Although the role of polyP in cancer cells was noticed earlier, including regulation of the mammalian target of rapamycin [6], the mechanistic implication of polyP in tumorigenesis remained unclear. Some authors demonstrated that treatment using exogenous polyP with a length of ~120 residues affects radio-sensitivity of H1299 cells and decreases cellular levels of ATP [39]. However, the mechanism of this treatment is not well understood, due to insufficient information about the uptake system for such a large negatively-charged polymer. Furthermore, it was documented that incubation of synthetic polyP with human osteogenic sarcoma cells led to the accumulation of ADP and ATP in the extracellular space of the cells [26]. In contrast to these findings, our results demonstrate that polyP endogenously produced by cancer cells may directly contribute to cell metabolism – as a novel phosphate energy source. Since cancer cells *in-vivo* encounter brief periods of starvation, which frequently occur as a result of insufficient tumor vascular supply [40] it is plausible that the cells employ opportunistic ways of obtaining necessary energy sources, including utilization of polyP, during those periods.

In contrast to bacteria, the triggering mechanisms and the corresponding eukaryotic pathways responsible for the synthesis and degradation of polyP have been largely unknown [21, 41, 42]. We anticipate that future studies will reveal the direct link between polyP accumulation, cellular bioenergetics and tumorigenesis. If so, a new field of human polyP metabolism regulation will emerge, providing the opportunity to exploit polyP as a targetable cancer-specific energy source.

## MATERIALS AND METHODS

### Cell culture

Previously described Radial Glial cell line LC26-10R and BTIC line LC26-RTL(4) (generated from LC26-10R) [29] were maintained in the exponential growth phase by passaging them every 3 days with growth medium Knockout DMEM/F-12 (Gibco, 12-660012) supplemented with 2% Stempro Neural Suppliment (Gibco, A1050801), 1% Hyclone Antibiotic/Antimycotic (GEHealthcare Life Sciences, SV30079.01SV3007901), 1% Glutamax (Gibco, 35-050-061) and 10 ng/mL bFGF (Sigma, GF003-AF) in laminin (Invitrogen, 23017015) coated 35 mm culture dish in CO_2_ incubator at 37°C.

### Mitochondrial staining

Mitochondria were stained with 50 nM of MitoTracker Red (Life Tech, M7512) in culture media for 15 minutes in growth conditions. Excess stain was washed with PBS and cells were fixed with 4% paraformaldehyde in PBS at room temperature for 30 minutes, followed by PBS wash.

### PolyP detection

For specific detection of polyP we used a method designed by Dr. Saito and colleagues [43]. This approach is based on tagging polyP with an engineered peptide, containing a polyP-binding domain (PPBD) from PPX, and an Xpress-tagged epitope, readily recognized by anti-Xpress-IgG. Briefly, cells were fixed with 4% paraformaldehyde, washed with PBS, and equilibrated with TBS (50 mM Tris, 0.15 mM NaCl, pH 8.3). The cells were permeabilized with 1 mL of 0.1% Triton X-100 in TBS for 20 minutes at room temperature in the presence of 0.2 mM MgCl_2_, followed by 2 washes with TBS. Blocking was done with 3% gelatin in TBS in the presence of 0.2 mM MgCl_2_ for 1 hour at 30°C, 3 rpm shaking. After blocking, the cells were incubated with 20 μg/mL of Polyphosphate binding domain tagged with Xpress-epitope (PPBD) in blocking buffer with 0.2 mM MgCl_2_ overnight at 30°C. The recombinant PPBD protein was expressed in *E. coli* TOP10 and purified by column chromatography as described previously [43]. Subsequently, the cells were washed twice with TBS following incubation with 2 μg/mL anti-Xpress antibody (Life technologies, 46-0528) in blocking buffer with 0.2 mM MgCl_2_ overnight at 30°C. Unbound antibody was washed with TBS. Anti-mouse Alexa flour-488 conjugated secondary antibody (Invitrogen, A21202) was used against Xpress antibodies in blocking buffer at 30°C for 1 hour. The cells were washed 3 times (the first two washes with 0.05% Triton X-100 in TBS, final wash with TBS). The chambers were removed and a coverslip was mounted on the slide with mounting media. The cell images were obtained using an Olympus BX61 confocal microscope under 60X magnification with appropriate fluorescent filters. The fluorescence intensity of the individual cells from 5 microscopic fields for each treatment were analyzed using ImageJ software. Average readings were obtained from at least from 7 cells for each condition.

### PolyP extraction

PolyP was extracted from equal amounts of LC26-10R or LC26-RTL(4) cells (15 × 10^6^ cells from each cell type) in three steps: (S1) Isolation of acid soluble short-chain polyP; (S2) Isolation of long-chain soluble polyP; (S3) Isolation of long-chain insoluble polyP, as described previously [33]. Extracts S1, S2 and S3 were combined and subjected to RNAse A (Invitrogen, 1209102) and DNAse I (Qiagen, 79254) treatments. Proteins were removed by extraction with an equal volume of phenol-chloroform followed by 3 successive extractions with chloroform. Polyphosphates precipitated by addition of 0.2 M final concentration of Tris-HCl, pH 7.6, and 2 volumes of acetone. The final pellet was resuspended in 30 μL of water. PolyP was detected and quantified on native-PAGE. Ten percent native-PAGE gel was pre-run at 200 V for 30 minutes. Samples were mixed with 6X loading buffer (6X TBE, 15% Ficoll, 0.025% xylene cyanol FF) before loading into the gel. With samples loaded, the gel was run in 1X TBE at 150 V for 1.30 hours in Mini-PROTEAN Tetra Vertical Electrophoresis Cell from Bio-Rad. Gels were stained with 0.05% w/v toluidine blue in fixative solution (25% methanol and 5% glycerol in water) and destained overnight with fixative solution. Synthetic PolyP (130) (kindly provided by Dr. Toshikazu Shiba, Regenetiss, Japan) was used as a loading control. The band intensities were analyzed using ImageJ software.

### Immunohistochemistry

After deparaffinization and antigen retrieval 4 μm thick formalin-fixed paraffin embedded (FFPE) tumor tissue sections were used for immunohistochemical analyses as well as TMAs purchased from US Biomax (MC484, GL1001a). All sections were blocked in Peroxidazed1 and Background Sniper for 15/10 min (Biocare medical, PX968 and BS966 respectively) and in Blocking buffer (3% gelatin in TBS, pH 8.3 containing 0.2 mM MgCl_2_) for 1 hour at 30°C. After blocking, the sections were incubated overnight at 30°C with PPBD in blocking buffer (1:40), washed 5 times with TBST for 40 minutes following incubation with Anti-Xpress antibody (Invitrogen, 46-0528, 1:1000) in Blocking buffer for 1 hour at 30°C. After two washes in TBST, MACH4 Mouse Probe and HRP-Polymer (Biocare medical, M4U534) were applied for 20 minutes each and color was developed with 3,3′-diaminobenzidine substrate (ThermoFisher Scientific Inc.). The sections were counterstained with hematoxylin.

### Starvation

Cells were cultured in 4-chambered microscopic slides (PEZGS0416, Millipore) in complete growth medium until they were 70% confluent, as described in “Cell culture” section. The starvation or glucose deprivation conditions were achieved by removal of Serum-free Stempro Neural Supplement, which has insulin as one of its key components. Even though Knockout DMEM/F-12 medium has high glucose content, removing insulin from the growth medium significantly decrease glucose intake by the cells. The cells grown in the complete growth medium, in parallel with the starved cells, were used as the control of 3 hr or 6 hr starvation experiments, which were conducted separately (Figure 3A). The slides were processed for polyP detection (as described above).

### ATP depletion

Cells were cultured in 4-chambered microscopic slides (PEZGS0416, Millipore) in complete growth medium until they were 70% confluent, as described in “Cell culture” section. To inhibit the ATP production, the cells were grown in Knockout DMEM/F-12 without Stempro Neural Supplement for 3 or 6 hours in the presence of 20 μM glycolytic inhibitor, iodoacetic acid (IAA) (Sigma, PI35603) and 20 μM mitochondrial ATP-synthase inhibitor, oligomycin (Alfa Aesar, J61898). The cells grown without Stempro Neural Supplement for 3 or 6 hours, in parallel with the starved cells treated with IAA and/or oligomycin, were used as the control of 3 hr or 6 hr ATP depletion experiments, which were conducted separately (Figure 3C and 3E). The slides were processed for polyP detection (as described above).

### Cell viability assay

LC26-10R and LC26-RTL(4) cells were cultured in 96-well plates in complete growth medium until they were 70% confluent, before conducting the starvation or ATP depletion experiments (as described above). After the starvation or ATP depletion experiments, the cells were washed with PBS and MTT (3- [4,5-dimethylthiazol-2-yl]-2,5-diphenyl tetrazolium bromide) (Acros Organics, 158990010) at a concentration of 0.5 mg/mL added to the cells in complete growth medium and incubated in growth conditions in the dark for 3 hours. The medium was removed and formazan crystals formed by the cells were dissolved using 50 μL of DMSO/ well. The absorbance values were measured using a microplate reader (Bio-Rad) at 570 nm (Figure 3B and 3D).

Cell survival of H1299 and HEK-293 cells (obtained from ATCC) was assayed by measuring the conversion of the yellow, water-soluble MTS [3-(4,5-dimethylthiazol-2-yl)-2,5-diphenyl tetrazolium bromide] into blue, water-insoluble formazan (Promega, Madison, WI). Cancer cells and HEK-293 were transfected with plasmid pPPX1 carrying *S. Cerevisiae* exopolyphosphatase ppx1gen using the Effectene reagent (Qiagen, Chatsworth, CA, USA). The next day cells were plated onto 96-well plates (1 × 10^4^ cells/well). After 3 days of growth at 37°C, the viability of the cells was assayed by incubation with MTS reagent for 2.5 hours at 37°C. The absorbance of the converted dye was measured at a wavelength of 490 nm with background subtraction at 655 nm using a microplate reader (Bio-Rad). In this assay, the value obtained from the cells transfected with empty vector was considered 100% viable.

### mitoGFP and mitoPPX expression

Cells were transiently transfected with pAcGFP1-Mito (Takara Bio) and pcDNA of mito-GFP-PPX using the Effectene reagent (Qiagen, Chatsworth, CA, USA) under serum-free conditions for 6 h in 4-chamber microscopic slides (PEZGS0416, Millipore). 48 and 72 h post-transfection, the cells were fixed with 4% paraformaldehyde followed by DAPI staining. The coverslips were mounted on slides using mounting media. Images of ten microscopic fields per well were obtained using a fluorescence microscope. Ten microscopic images under 40× magnification were obtained for each condition using a fluorescence microscope. The average percentages of GFP positive cells were calculated against the total number of DAPI stained cells in each microscopic image. The fold change in the percentage of GFP positive cells at 72 h was plotted against 42 h.

### Mg^2+^-imaging of radial glial (LC26-10R) and cancer (RTL-4) stem cells

Fluorescence measurements of the intracellular Mg^2+^ were performed on LC26-10R and LC26-RTL(4) cells transiently expressing Mito-GFP-PPX or AcGFP1.Mito (control) upon IAA (20 μM) and oligomycin (20 μM) application. HEPES-buffered salt solution (HBSS) containing (in mM units): 156 NaCl, 3 KCl, 2 MgSO_4_, 1.25 KH_2_PO_4_, 2 CaCl_2_, 10 glucose, and 10 HEPES, pH 7.35 (adjusted with NaOH) was used as an extracellular solution in ratio-metric [Mg^2+^] measurements [22]. Cells were incubated with 5 μM MagFura in the presence of 0.01% Pluronic for 30 minutes at room temperature, rinsed once with PBS following 10 min. wash using the imaging buffer. The measurements were performed using a Photon Technology International (Birmingham, NJ) imaging system mounted on an Zeiss-AXIO observed D1 microscope, equipped with Ratiomaster 5 imaging system (Photon Technology International) and a Cool-snap HQ2 (Roper) camera.

### Fluorescence microscopy after DAPI staining

H1299 lung cancer, U251 glioma, or HEK-293 cells (all cells obtained from ATCC) (with or without transient expression of PPX1) were grown on 25 mm round glass coverslips and fixed with 2 ml of 4% paraformaldehyde in PBS at room temperature for 30 minutes. The cells were washed twice with PBS (2 mL) and incubated with 7.2 μM 4′-6-diamidino-2-phenylindole (DAPI) for 30 minutes. Then cells were washed three times (2 mL each) with PBS, washed once with distilled water, and mounted on a glass slide with mounting media. The cell images were obtained using confocal microscopy (Olympus BX61 Fluoview, Minneapolis, MN) at 60× magnification.

Bars display mean ± S.D. One-way ANOVA (*p* < 0.05) was performed using OriginPro^®^.
